# Epidemic forecasting based on mobility patterns: an approach and experimental evaluation on COVID-19 Data

**DOI:** 10.1007/s13278-022-00932-6

**Published:** 2022-08-18

**Authors:** Maria Pia Canino, Eugenio Cesario, Andrea Vinci, Shabnam Zarin

**Affiliations:** 1 University of Calabria, Rende (CS), Italy; 2 ICAR-CNR, Rende (CS), Italy; 3 Monmouth University, West Long Branch, NJ USA

**Keywords:** COVID-19, Epidemic forecasting, Predictive models

## Abstract

During an epidemic, decision-makers in public health need accurate predictions of the future case numbers, in order to control the spread of new cases and allow efficient resource planning for hospital needs and capacities. In particular, considering that infectious diseases are spread through human-human transmissions, the analysis of spatio-temporal mobility data can play a fundamental role to enable epidemic forecasting. This paper presents the design and implementation of a predictive approach, based on spatial analysis and regressive models, to discover spatio-temporal predictive epidemic patterns from mobility and infection data. The experimental evaluation, performed on mobility and COVID-19 data collected in the city of Chicago, is aimed to assess the effectiveness of the approach in a real-world scenario.

## Introduction

**Reference Context.** An epidemic is the rapid spread of a disease to a large number of people in a given population in a short period of time. Usually, it poses a serious threat not only to public health but also to healthcare institutions and economies as a whole (Schwabe et al. 2021). In the last two years, the whole world has experienced huge issues and problems due to COVID-19, which, as of March 28, 2022, has been responsible for more than 400 million reported cases (source data: Johns Hopkins University Center for Systems Science and Engineering Dong et al. 2020), thus resulting as one of the worst pandemics in history.

To control the spread during an epidemic, decision-makers in public health need accurate predictions of future case numbers. This allows for early interventions and, on top of that, is important for near real-time resource planning, so that hospital needs can be satisfied without exceeding their capacities (Ferguson et al. 2020). To do that, several explanatory models have been designed and studied for epidemic forecasting, aimed at modeling virus spreading dynamics and evolutions.

**Motivations and Contributions.** As infectious diseases are spread through human-human transmissions, spatio-temporal mobility data can play a fundamental role to enable epidemic forecasting. In fact, mobility data can be derived from movements among several locations of urban and/or sub-urban areas and, differently from static data, have the added value of dynamically tracing the underlying trajectories, providing a more comprehensive description of human-human interactions. Moreover, there can be some areas, like the touristic ones, in which there are more users (tourists) prone to produce and share contexts, data and messages by social media, so data can be integrated by different sources (Cesario et al. 2017). In particular, the underlying hypothesis of our work is that human mobility can strongly affect virus spreading, thus the extraction of mobility patterns among congested areas can enable the prediction of spatio-temporal epidemic patterns. More specifically, the identification of regions that are both infection hotspots (thus involved in high density of infection cases ) and mobility hotspots (thus involved in mobility patterns) is a crucial step of the process to identify how mobility patterns can also play a role of epidemic patterns.

This paper presents the design and implementation of an approach based on spatial analysis and regressive models to discover *spatio-temporal predictive epidemic models* from mobility and infection data. The algorithm is composed of several steps. First, infection hotspots (urban regions more densely affected by infection events with respect to others) and mobility hotspots (urban regions more densely visited by mobility traces) are detected. Then, mobility patterns among mobility hotspots are discovered. On the basis of such knowledge, epidemic hotspots (infection hotspots whose spatial overlap with mobility hotspots involved in mobility patterns is greater than a given threshold) and epidemic patterns are generated from the mobility patterns, by selecting those that have epidemic hotspots involved as both source and destination areas. Finally, the approach extracts a specific epidemic forecasting model for each epidemic hotspot, analyzing the infection data of the epidemic hotspots involved in mobility patterns.

**Plan of the Paper.** The rest of the paper is organized as follows. Section 2 reports the most important approaches in the virus spread forecasting literature and the most representative projects in that field of research. Section 3 outlines the problem statement and the goals of our analysis. Section 4 presents the proposed approach, based on spatial analysis and regressive models to discover *spatio-temporal predictive epidemic models* from mobility and infection data. Section 5 describes the experimental evaluation, performed on a real-world case study concerning the city of Chicago, aimed at showing the most significant mobility patterns among hotspots, the epidemic hotspots, and effective predictive models, which can estimate the number of epidemic events that are likely to happen in the future. We also performed a comparative analysis of our results with respect to a base-line approach (i.e., auto-regression algorithm). Finally, Sect. 6 concludes the paper and plans future research work.

## Related works

Forecasting virus spread is very important in the public health domain, because it can support decision makers to anticipate epidemic trends and thus to optimize public resources’ allocations. This is undoubtedly a challenging task and, since 2019, it has been experiencing a large effort of virologists, epidemiologists and data scientists to predict COVID-19 diffusion. In this section we briefly review the most representative research works in this field, grouped in two main categories: *mechanistic/stochastic models* and *AI/data-driven models*. Then, we report a critical comparison (on the basis of some specific features) among the method we developed and state-of-art solutions.

**Mechanistic and stochastic models.** Traditionally, the models for epidemic forecasting proposed in the literature can be distinguished in two categories: mechanistic models and stochastic models (Schwabe et al. 2021). Mechanistic models explicitly utilize epidemiological theory and empirical evidence, while stochastic models mainly rely on the predictive power of data. A type of mechanistic model is the compartmental model, which has been exploited in some research works on COVID-19 (Bertozzi et al. 2020; Chang et al. 2021). A compartmental model is based on the assumption that each individual belongs to some compartment (e.g., susceptible, infected, recovered) and has a certain probability of transitioning to another compartment. The probability of a transition can be either derived from case data or modeled as being dependent on additional predictors. Stochastic models are based on probability distributions to predict the evolution of the events. In Reinhard and Frank (2020) a Gaussian model is used as stochastic model to predict the peak of COVID-19 cases, while in Bertozzi et al. (2020) an exponential model is exploited to predict the disease spread in the early stages.

**AI and data-driven models.** Recently, artificial intelligence (AI) methods and data-driven approaches integrating mobility, social networks, web search, and air quality data have been proposed in literature. Interestingly, in Comito and Pizzuti (2022) is provided a comprehensive review of methods, algorithms, applications, and emerging AI technologies that can be utilized for forecasting and diagnosing COVID-19. In particular, the purpose of this review is to investigate and discuss an extensive collection of papers with the aim of giving an overview of how AI can help fighting COVID-19 pandemic. In Yabe et al. (2022) an approach based on human mobility trajectories (collected as GPS traces) and web search queries (with common user identifiers) has been proposed, to predict COVID-19 hotspot locations beforehand. More specifically, a web search query analysis is conducted to identify users with a high risk of COVID-19 contraction, and social contact analysis was further performed on the mobility patterns of these users to quantify the risk of an outbreak. The approach has been empirically tested using data collected from users in Tokyo, Japan, to predict COVID-19 hotspot locations 1-2 weeks beforehand. In Schwabe et al. (2021) a model for epidemic forecasting based on mobility data, called mobility marked Hawkes model, has been proposed. This model consists of (*i*) a Hawkes process that captures the transmission dynamics of infectious diseases, (*ii*) a Poisson regression model which modulates the rate of infections (thus accounting for how the reproduction number *R* varies across space and time), and (*iii*)a correction procedure taking into account new cases seeded by people traveling between regions. This model has been used to predict the COVID-19 epidemic in Switzerland, over different forecast horizons between 5 and 21 days. The paper (Mokhlesur Rahman et al. 2021) presents a review study aimed at analyzing interactions among the COVID-19 pandemic, lockdown measures, human mobility, and air quality. In particular, the paper shows that urban form, people’s socioeconomic and physical conditions, social cohesion, and social distancing measures significantly affect human mobility and COVID-19 transmission. The study also noticed that lockdown measures applied during COVID-19 significantly improved air quality by reducing the concentration of air pollutants, which in turn improved the COVID-19 situation by reducing respiratory-related sickness and deaths of people. In Ilin et al. (2021) a study is presented showing how public available data on human mobility (collected by Google, Facebook, and other providers) can be used to evaluate the effectiveness of non-pharmaceutical interventions (NPIs) and forecast the spread of COVID-19. The approach has been evaluated using local and regional data from China, France, Italy, South Korea, and the United States, and has been applied to provide 10-day forecasts of COVID-19 cases. The paper (Comito 2021) presents a methodology based on Twitter data analysis that combines peak detection and clustering techniques to inspect how information about the COVID-19 epidemics spread in the US. To this purpose, the objectives are to identify the key terms and features used in the tweets, the interest in the COVID-19 topics, together with the evolution of the discussion all over the US. Spacetime features are extracted from the tweets and modeled as time series. After that, peaks are detected from the time series, and peaks of textual features are clustered based on the co-occurrence in the tweets.

**Comparative analysis among the approaches.** Now, we report a critical comparison of the proposed approach and some other solutions proposed in the literature. Specifically, the comparison of the different approaches has been made on the basis of some specific features (i.e., hotspot detection approach, hotspot shapes, forecasting approach), as summarized in Table 1 and detailed in the following:

*Hotspot detection approach*. This feature differentiates the algorithms on the basis of the approach used to detect spatial (epidemic) hotspots. Our approach uses spatial density-based clustering to detect interesting hotspots, while the approaches presented in Schwabe et al. (2021); Ilin et al. (2021) rely on pre-defined regions (phone cellular grid cells and regular square regions, respectively). On the other side, the algorithm described in Comito (2021) exploits a topic-based clustering approach to detect groups of textual features on the basis of their co-occurrence in the tweets. Finally, the approach presented in Yabe et al. (2022) adopts a web search-based approach to identify Covid-19 hotspots, where a user is identified as a high risk user if he/she had more than *k* (pre-defined threshold value) COVID-19 related web search sessions.

*Hotspot shapes*. This feature takes into account the shape of the detected hotspots, which is relevant to assess the ability of the detection approach in identifying any possible area, regardless of the shape. Our approach is capable of detecting regions of any shape (e.g., circular, rectangular, irregular), while the works described in Schwabe et al. (2021); Ilin et al. (2021) deal with only specific region shapes. The algorithm described in Comito (2021) partitions the geographic area according to the US state borders and performs the analysis at state-granularity shapes. Finally, the approach presented in Yabe et al. (2022) presents the experimental evaluation by splitting the geographic area in both $1Km \times 1Km$ and $125m \times 125m$ grid cells in Tokyo metropolitan region, thus considering hotspots with specific square shapes.

*Regression approach*. This feature classifies the approaches on the basis of the regression methodology used to forecast new COVID-19 case numbers. Specifically, our approach exploits the LSTM method, while the methodologies described in Schwabe et al. (2021); Ilin et al. (2021) exploit Poisson regression and polynomial regression, respectively. On the other side, the algorithm described in Comito (2021) adopts two forecasting models, one expressed as a specific auto-regression problem and the other based on a Bayesian approach. Finally, the approach presented in Yabe et al. (2022) adopts a social contact index (SCI) and a time-lagged cross-correlation analysis to predict the number of new cases.

From the above comparative evaluation, we can summarize the main differences the proposed approach exhibits with respect to the other ones proposed in literature. First, it detects infection and mobility hotspots as they emerge from real infection and mobility data, without relying on predefined static subdivisions of the spatial area (as done in Schwabe et al. (2021) and Ilin et al. (2021)), and thus enabling the detection of hotspots of any shape. Second, our approach relies on mobility data to predict further epidemic evolutions among several locations of urban and/or suburban areas. Thus, it is based on a comprehensive data-driven spatio-temporal knowledge of human-human interactions, rather than exploiting stochastic and probabilistic models to forecast future infection cases.

**Table 1:** Comparison of several approaches proposed in literature.

Approaches	Hotspot Detection	Hotspot Shape	Regression approach
*The proposed approach*	*density-based clustering*	*any shape*	*LSTM*
*Reference* (Schwabe et al. 2021)	*phone cellular cells*	*grid cells*	*Poisson*
*Reference* (Ilin et al. 2021)	*pre-defined square regions*	*square regions*	*polynomial*
*Reference* (Comito 2021)	*topic-based clustering*	*state borders*	*auto-regression and Naive Bayes*
*Reference* (Yabe et al. 2022)	*web search-based*	*grid cells*	*SCI (social contact index)*

## Problem definition and goal

We begin by fixing a proper notation and giving some definitions to be used throughout the paper.

**Timestamp List.** Let $T=<t_1,t_2,\ldots ,t_H>$ be an ordered timestamp list, such that $t_h<t_{h+1}, \forall _{ 0<h<H}$, and where all $t_h$ are at equal time intervals (e.g., every minute, hour, day).

**Infection Data.** Let ${{\mathcal {I}}}{{\mathcal {D}}}$ be a dataset collecting infection data instances, ${{\mathcal {I}}}{{\mathcal {D}}}=\{ID_1,ID_2,\ldots ,ID_M\}$, where each $ID_i$ is a data tuple $<n_i, lat_i, long_i, t_i>$ described by the following features: $n_i$ is the number of infection cases (i.e., number of positive cases) detected, $lat_i$ and $long_i$ are the *latitude* and *longitude* of the place the infection event has occurred, $t_i$ (with $t_i \in T$) is the observation timestamp.

**Mobility Data.** Let ${{\mathcal {M}}}{{\mathcal {D}}}$ be a dataset collecting mobility data instances, ${{\mathcal {M}}}{{\mathcal {D}}}=\{MD_1,MD_2,\ldots ,MD_N\}$, where each $MD_i$ is a spatio-temporal trajectory instance $MD_i=<(lat_{i1},log_{i2},t_1), \ldots , (lat_{iH},long_{iH},t_H)>$, where each triple $(lat_{ih},long_{ih},t_h)$ indicates that an object of the trajectory $MD_i$ is in the position $lat_{ih},long_{ih}$ at time $t_h$ (with $t_h \in T$).

**Infection Hotspots.** Let ${{\mathcal {I}}}{{\mathcal {H}}}$ be a set of infection hotspots, ${{\mathcal {I}}}{{\mathcal {H}}}=\{IH_1,IH_2,\ldots ,IH_N\}$, where each $IH_i$ is an area where infection events of a specific disease occur with an higher density with respect to other neighbor areas.

**Mobility Hotspots.** Let ${{\mathcal {M}}}{{\mathcal {H}}}$ be a set of mobility hotspots, ${{\mathcal {M}}}{{\mathcal {H}}}=\{MH_1,MH_2,\ldots ,MH_N\}$, where each $MH_i$ is an area that is more densely visited by the object’s trajectories with respect to other areas.

**Mobility Patterns.** Let ${{\mathcal {M}}}{{\mathcal {P}}}$ be a set of (frequent) mobility patterns, ${{\mathcal {M}}}{{\mathcal {P}}}=\{MP_1,MP_2,\ldots ,MP_N\}$, where each $MP_i$ is a sequential pattern of two mobility hotspots, in the form $(MH_{t_s}^{j_s} \rightarrow MH_{t_d}^{j_d})$ with time constraints $t_r < t_s$. The block on the left $MH_{t_s}^{j_s}$ is the source hotspot, while the block on the right $MH_{t_d}^{j_d}$ is the destination hotspot of the pattern. Mining of sequential patterns consists of mining the set of subsequences occurring with a support *sup* higher than a given minimum threshold $sup_{min}$.

**Overlapping infection hotspots.** Let $oih_{{{\mathcal {I}}}{{\mathcal {H}}}}(MH_i)$ be the set of infection hotspots $IH_j$ having a spatial overlap with $MH_i \in {{\mathcal {M}}}{{\mathcal {H}}}$ higher than a given threshold $\delta _{min}$, and thus $oih_{{{\mathcal {I}}}{{\mathcal {H}}}}(MH)=\{ IH \mid IH \in {{\mathcal {I}}}{{\mathcal {H}}} \wedge \delta (IH,MH)\ge \delta _{min} \}$, for all MH in ${{\mathcal {M}}}{{\mathcal {H}}}$, and $\delta (\cdot ,\cdot )$ being a function computing the spatial overlapping percentage between its parameters.

**Epidemic Hotspots.** An epidemic hotspot is an infection hotspot $IH_j$ having a spatial overlap (higher than a given threshold $\delta$) with a mobility hotspot $MH_k$ and if $MH_k$, and thus ${{\mathcal {E}}}{{\mathcal {H}}}=\{ EH \mid EH \in {{\mathcal {I}}}{{\mathcal {H}}} \wedge \exists MH \in {{\mathcal {M}}}{{\mathcal {H}}} \mid EH \in oih_{{{\mathcal {I}}}{{\mathcal {H}}}}(MH) \}$.

Now, let us consider a future temporal horizon, $S=<t_w, t_{w+1}, \ldots>$, with $w>H$. The goal of the analysis is to find epidemic spread models for reliably predicting the number and location of new infection events at a given timestamp $t_w \in S$. More specifically, given the infection dataset ${{\mathcal {I}}}{{\mathcal {D}}}$ and the mobility dataset ${{\mathcal {M}}}{{\mathcal {D}}}$, our analysis aims at achieving the following goals:

1. discover a set ${{\mathcal {E}}}{{\mathcal {H}}}$ of *epidemic hotspots*, ${{\mathcal {E}}}{{\mathcal {H}}} = \{EH_1, \ldots , EH_K\}$, where a *epidemic hotspot* $EH_k$ is a spatial area both affected by higher density of infections than other areas and involved in frequent mobility patterns (as above described);
2. discover a set ${{\mathcal {E}}}{{\mathcal {P}}}=\{EP_1,EP_2,...\}$ of *epidemic patterns*, where each *EP* is a couple $(EH_s,EH_d)$, meaning that the infection trend of $EH_s$ influences the infection trend of $EH_s$.
3. extract a function $F_{spreading}:{\mathcal {S}}\rightarrow \mathcal {({{\mathcal {E}}}{{\mathcal {H}}},R)}$ that, given a timestamp $t_w \in S$, states the number of epidemic events (i.e., number of positive cases) $N \in {\mathcal {R}}$ that are predicted to happen in each *epidemic hotspot* $EH_i \in {{\mathcal {E}}}{{\mathcal {H}}}$ at the timestamp $t_w$.

## The proposed approach

This section describes the algorithm that we have designed to discover *spatio-temporal predictive epidemic models* from mobility and infection data. Specifically, Sect. 4.1 depicts the main steps of the proposed approach and its pseudo-code, whereas Sects. 4.2, 4.3, and 4.4 describe in details the procedures for infection and mobility hotspots detection, mobility patterns extraction and epidemic forecasting models training, respectively.

### Algorithm’s wokflow and pseudo-code

**Fig. 1:**
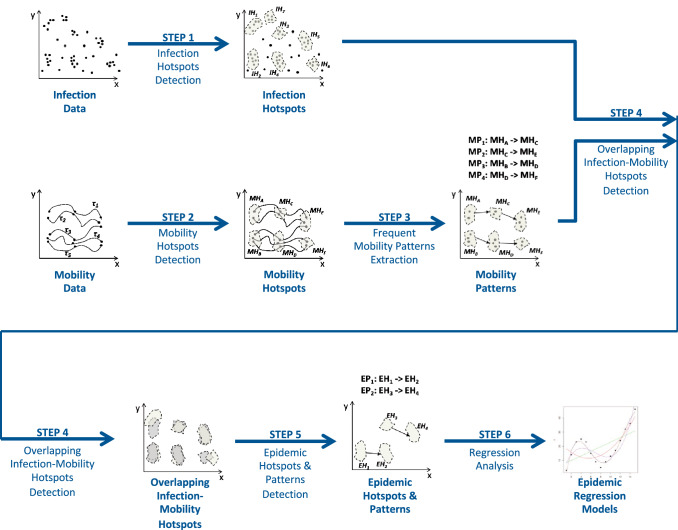
The Approach Workflow

The main workflow of the proposed approach is shown in Fig. 1, while the pseudo-code is reported in Algorithm 1. For the reader’s convenience, Table 2 reports the meaning of the main symbols used throughout the code.

The algorithm receives in input the infection and the mobility datasets (represented in the previously format described in Sect. 3), and returns a set of epidemic hotspots, epidemic patterns and epidemic regression models. The workflow is composed of six steps (see Fig. 1 and Algorithm 1), as described in the following.

**Step 1 and 2: Infection and Mobility Hotspots Detection. ** These two steps are aimed at detecting Infection and Mobility Hotspots from Infection and Mobility datasets, respectively. More specifically, Infection Hotspots are urban regions more densely affected by infection events with respect to others (thus, of interest for the further analysis), while Mobility Hotspots are urban regions more densely visited by mobility traces. This is done by running density-based clustering algorithm instances whose final result consists of N and M clusters (each corresponding to a detected dense region). The detected hotspots, whose number is automatically detected by the algorithm, can have different shapes and sizes. These two steps are performed by the DiscoverHotspots method on the Infection and Mobility datasets, respectively (lines 1 and 2, Algorithm 1).

**Step 3: Frequent Mobility Patterns Extraction. ** During this step a sequential pattern extraction algorithm on the detected mobility hotspots is executed, to discover frequent mobility patterns from them. The final mining model is a set of rules describing mobility relations between the movement of the users under investigation. For the sake of clarity, in this work we represent mobility patterns composed of one source and one destination hotspots. This is done by running the ExtractMobilityPatterns method (line 3, Algorithm 1).

**Step 4: Overlapping Infection-Mobility Hotspots Detection. ** This step is aimed at detecting epidemic hotspots, that is, infection hotspots whose spatial overlap with mobility hotspots involved in mobility patterns is higher than a given threshold $\delta _{min}$. Since our hypothesis is that mobility can strongly affect the infection spreading, then the identification of regions which are both infection hotspots (thus involved in high density of infection cases ) and mobility hotspots (thus involved in mobility patterns) is a crucial step of the process. The spatial overlap is calculated as the percentage of the overlapping area between the identified infection and mobility hotspots. In Algorithm 1 this step is implemented by the lines 4-11.

**Step 5: Epidemic Hotspots and Patterns Detection. ** On the basis of the overlapped hotspots detected, this step is aimed at detecting (*i*)epidemic hotspots and (*ii*)epidemic patterns. In particular, an epidemic hotspot is an infection hotspot that has a reasonable spatial overlap with a mobility hotspot involved in a mobility pattern. As epidemic hotspots are detected, epidemic patterns are generated from the mobility patterns, by selecting those having epidemic hotspots involved as both source and destination areas. The computation of the Epidemic Hotspots ${{\mathcal {E}}}{{\mathcal {H}}}$ and the Epidemic Patterns ${{\mathcal {E}}}{{\mathcal {P}}}$ is done by the lines 12-21 of Algorithm 1.

**Step 6: Epidemic Spread Forecasting. ** This step is aimed at extracting a specific epidemic forecasting model for each epidemic hotspot that is a destination of an epidemic pattern, by exploiting data from its sources. The training of the epidemic forecasting models is done by the lines 22-31 of the Algorithm 1.

In particular, for each epidemic hotspot $EH_i$, an Epidemic Time Series $ETS_i$ is extracted, by the function BuildEpidemicTSData (line 28) applied in its Source Epidemic Hotspots $SEH_i$ and the infection data *ID*. $ETS_i$ is a multivariate time series that aggregates the number of infections occurred in a time interval (e.g., each day) in $EH_i$ and in each source epidemic hotspot in $SEH_i$. Given the Epidemic Time Series $ETS_i$ related to $EH_i$, the DiscoverEpidemicModel (line 29) method learns a forecasting model for predicting the number of epidemic events in the future, inside the epidemic hotspot $EH_i$.
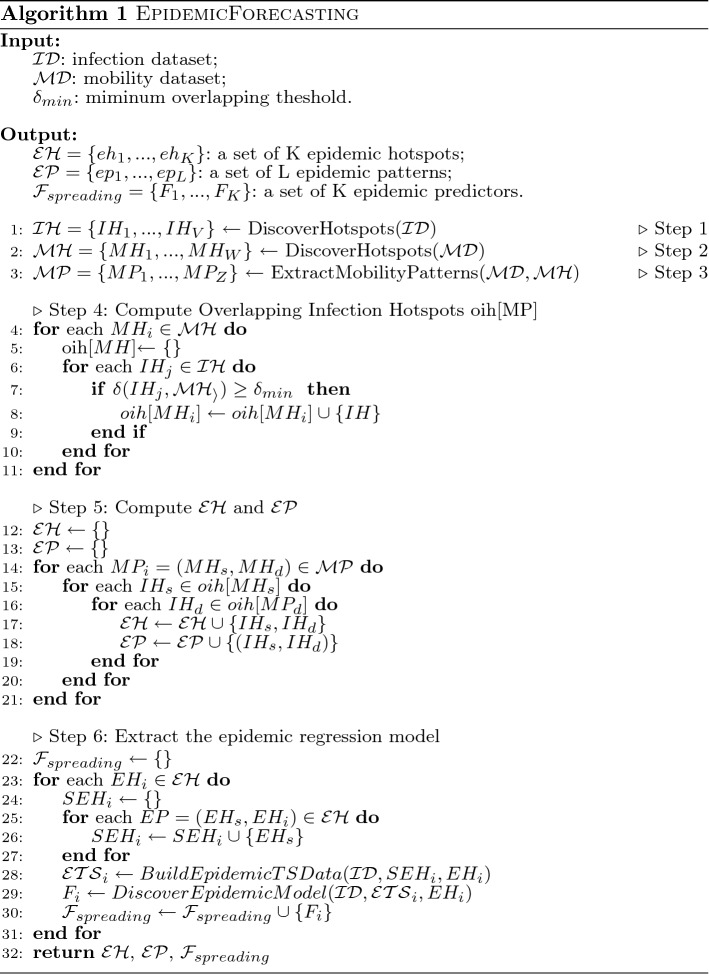

**Table 2:** Meaning of the main symbols used

Symbol	Meaning
$T=\{t_1, t_2,\dots ,t_H\}$	Timestamp list
${{\mathcal {I}}}{{\mathcal {D}}}=\{ID_1, ID_2, \dots , ID_M\}$	Infection data
${{\mathcal {M}}}{{\mathcal {D}}}=\{MD_1, MD_2, \dots , MD_N\}$	Mobility data
${{\mathcal {I}}}{{\mathcal {H}}}=\{IH_1, IH_2, \dots , IH_V\}$	Infection hotspots
${{\mathcal {M}}}{{\mathcal {H}}}=\{MH_1, MH_2, \dots , MH_W\}$	Mobility hotspots
${{\mathcal {M}}}{{\mathcal {P}}}=\{MP_1, MP_2, \dots , MP_Z\}$	Mobility patterns
*oih*[*MP*]	Set of the infection hotspots overlapping $MP_z \in {{\mathcal {M}}}{{\mathcal {P}}}$
${{\mathcal {E}}}{{\mathcal {H}}}=\{EH_1, EH_2, \dots , EH_K\}$	Epidemic hotspots
${{\mathcal {E}}}{{\mathcal {P}}}=\{EP_1, EP_2, \dots , EP_L\}$	Epidemic patterns
$\mathcal {ETS}=\{ETS_1, ETS_2, \dots , ETS_K\}$	Epidemic Time Series
$\mathcal {SEH}=\{SEH_1, SEH_2, \dots , SEH_K\}$	Source epidemic hotspots
${\mathcal {F}}_{spreading}=\{F_1, F_2, \dots , F_K\}$	Regression functions, one for each $EH \in {{\mathcal {E}}}{{\mathcal {H}}}$

### Detection of Infection and Mobility Hotspots

The DiscoverHotspots method (lines 1 and 2) performs a spatial clustering of the data set, where each cluster represents a hotspot of events. The density-based notion is a common approach for clustering, whose inspiring idea is that objects forming a dense region should be grouped together into one cluster. In our implementation, this task has been performed by applying DBSCAN (Ester et al. 1996), a popular density-based clustering algorithm that finds clusters starting from the estimated density distribution of the considered data. We have chosen the DBSCAN algorithm because it has the ability to discover clusters with arbitrary shape such as linear, concave, oval, etc. and (in contrast to other clustering algorithms proposed in the literature) it does not require the predetermination of the number of clusters to be discovered. Basically, the algorithm finds clusters with respect to the notion of density reachability among points: a point is directly density-reachable from another point if it is not farther away than a given distance ($\epsilon$) (i.e., is part of its neighborhood) and if it is surrounded by sufficiently many points (*minPts*). In the considered context, a cluster corresponds to an hotspot. Finally, DBSCAN requires the user to specify the radius of the neighborhood (i.e., $\varepsilon$) and the minimum number of objects it should have (i.e., *minPoints*), whose values affect size and density of the discovered clusters. Generally, an optimal setting of its parameters is complex to be achieved and requires specific techniques; however, this topic is outside the scope of this paper.

### Extraction of mobility patterns

The ExtractMobilityPatterns method (line 3) performs the detection of mobility patterns between two hotspots. We implemented this task by the T-Apriori algorithm (Cesario et al. 2017), an Apriori-based algorithm that extracts rules whose elements respect a monotonically increasing time order (i.e., the timestamps of the antecedents are chronologically previous of those appearing in the consequent). The final result is a set of sequential patterns between two mobility hotspots, in the form $SH \rightarrow DH$. The block on the left *SH* is the source hotspot, while the block on the right *DH* is the destination hotspot of the pattern. The algorithm requires the user to specify the $sup_{min}$ and $conf_{min}$ thresholds, and it discovers the set of sub-sequences occurring with a support $sup\ge sup_{min}$ and a confidence $conf\ge conf_{min}$.

### Discovery of epidemic forecasting models

The *DiscoverEpidemicModel* method (line 29 in Algorithm 1) performs the training of an epidemic forecasting model given a multivariate time series that aggregates the number of infections that occurred in a time interval in a target epidemic hotspot and its sources previously identified. This task can be done through different time series forecasting approaches, such as Simple Exponential Smoothing (SES) and Auto-regressive models (ARIMA). In this work we exploit Recurrent Neural Nnetworks, and in particular Long-Short Term Memory (LSTM) neural networks.

LSTMs (Hochreiter and Schmidhuber 1997; Sak et al. 2014) have been proved to be effective in forecasting tasks, due to their capability of processing sequences. They are also widely used in speech recognition and language translation. An LSTM neural network is a particular kind of recurrent neural network (RNN) and thus admits feedback connections. LSTMs are characterized by the presence of a block of memory cells, which are special structures permitting the network to remember values over arbitrary time intervals. Four gates control a cell’s information flow and state: an input gate, an output gate, a forget gate, and a cell gate. The following equations define the behavior of an LSTM unit:

1 $$\begin{aligned} \begin{array}{l} i_t=\sigma (W_{ii}x_t + b_{ii}+W_{hi}h_{t-1}+b_{hi})\\ f_t=\sigma (W_{if}x_t + b_{if}+W_{hf}h_{t-1}+b_{hf})\\ g_t=\tanh (W_{ig}x_t + b_{ig}+W_{hg}h_{t-1}+b_{hg})\\ o_t=\sigma (W_{io}x_t + b_{io}+W_{ho}h_{t-1}+b_{ho})\\ c_t=f_t \odot c_{t-1} + i_t \odot g_t \\ h_t=o_t \odot \tanh (c_t) \end{array} \end{aligned}$$

where $h_t$ is the hidden state at time *t*, $c_t$ is the cell state at time *t*, $x_t$ is the input at time *t*, $i_t$, $f_t$, $g_t$, $o_t$ are the input, forget, cell, and output gates, respectively. $\sigma$ is the sigmoid activation function, and $\odot$ is the Hadamard product. *W* and *b* are the weights to be learned during the LSTM optimization. It is worth noting that the recurrent nature of LSTM is given as both $c_t$ and $h_t$ depend on $c_{t-1}$ and $h_{t-1}$, which are their values at the previous step.

In this work, we exploited a neural network embedding an LSTM layer. The models are trained by exploiting the well-known ADAM optimizer (Kingma and Ba 2014).

## Analysis and experimental results

**Fig. 2:**
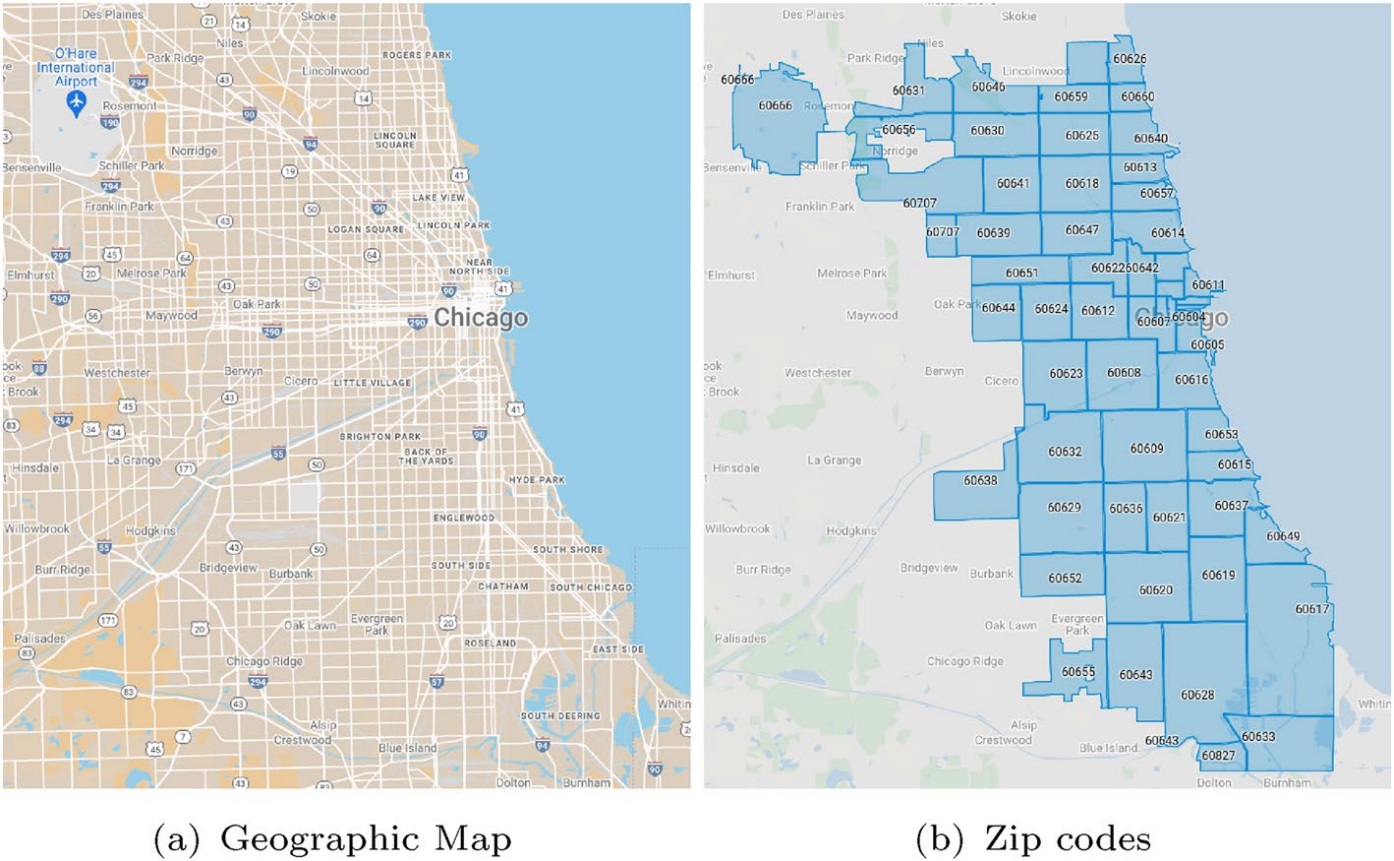
Map of Chicago and its zip codes

To evaluate the performance and the effectiveness of the approach described above, we carried out an experimental analysis by performing different tests in a real-world case study concerning the city of Chicago, whose map and zip codes are shown in Fig. 2. The goal of our analysis comprises detecting the most significant mobility patterns among hotspots, the epidemic hotspots and effective predictive models, which can estimate the number of epidemic events that are likely to happen in the future. We also performed a comparative analysis of our results with respect to a baseline, achieved by auto-regression. In the following subsections we describe the main issues of our analysis: data description and gathering (Section 5.1), the regressive model training, the detection of epidemic hotspots and mobility patterns (Section 5.2), training and testing of the forecasting model (Section 5.3), the experimental evaluation of the model on the test set and a comparative analysis with an auto-regression model (Section 5.4).

### Data description

The data that we used to train the models and perform the experimental evaluation have been gathered from *Chicago Data Portal* [18] and *Observable Web Portal* [19], two public data search and exploration platforms hosting datasets related to urban and extra-urban environments. In particular:

- *Mobility data* have been gathered from the *’Public Passenger Vehicle Licenses’* dataset housed on the *Chicago Data Portal*, a real-life collection of trajectories traced by public passenger vehicles (i.e., licensed taxicabs, liveries, ambulances, medicars, charter-sightseeing buses, horse-drawn carriages, and pedicabs) in the city of Chicago. Each trajectory is described by source place and destination place.
- *Infection data* have been retrieved from the *’Historical Illinois COVID-19 ZIP code data’*, a dataset populated by daily snapshots of Illinois Department of Health counts by ZIP code^1^. This dataset has been retrieved as open data by the *Observable Web Portal*, a Web framework which gives public access to several datasets storing urban data^2^. In particular, for our analysis we have collected infection data from April 18, 2020 to December 20, 2021. Each infection event is described by several attributes (i.e., zip code, date, cumulative number of tested, cumulative number of positive cases, etc.).

Mobility data have been analyzed to discover mobility patterns and epidemic hotspots, while the infection data have been analyzed to discover predictive models for epidemic spread forecasting.

**Fig. 3:**
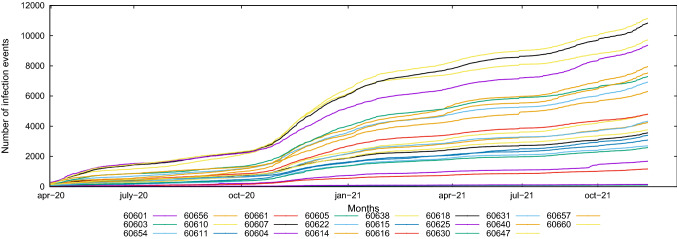
Cumulative number of infection cases vs time, for each zip code

Figure 3 shows a preliminary view of the infection data collected, which provides some hints about data trends and distribution. In particular, it shows the time plot of the number of observed infection data for each zip code, in which the cumulative number of infections (positive cases) is plotted versus the time of observation. From the plot, we see that the occurrence of infection is almost stable during Spring 2020 and Summer 2020, strongly increases in late Autumn 2020 and Winter 2020-2021, again achieving a stable trend during Spring and Summer 2021, and returning to rise in Autumn 2021.

**Fig. 4:**
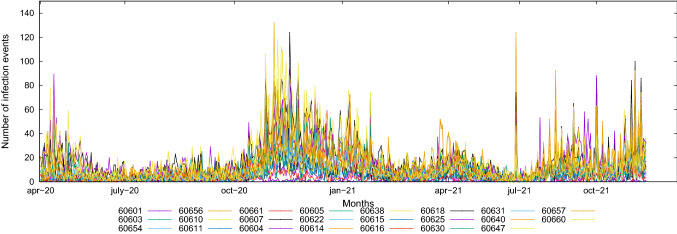
Number of infection cases vs time, for each zip code

Starting from the collected cumulative data, we computed the daily number of infected cases by performing the difference between two consecutive measurements, for each zip code. We plot the daily data in Fig. 4, which reveals some additional interesting features about data trends and distribution. First, it is evident that the number of detected infections is very unstable among the observation time, showing an *high spread* of the time series. Second, a *seasonal pattern* is clearly observable, that shows a multi-wave pattern in the data. From the plot, we see that the number of positive cases increased in April 2020, decreased during the Summer 2020, increased in late Autumn 2020 and during the Winter 2020-2021, decreased again during the Spring 2021 (with a peak in April 2021) and the Summer 2021, and returning to rise again during the Autumn 2021.

**Fig. 5:**
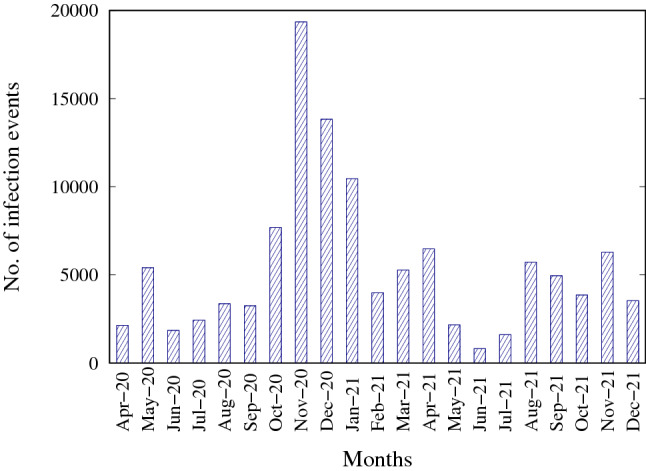
Number of infection cases by month in the whole city area

A clearer view of the seasonality hidden in the data can be seen in Fig. 5, which shows the distribution of the number of positive cases by month in the whole city area, that is, cumulated over all the zip codes. The histogram shows that the number of infection events varies significantly between different periods of the year. In particular, the number of infection events is highest in November 2021, and lowest in June 2021.

### Detection of Epidemic Hotspots and Epidemic Patterns

Before the analysis of the trajectories, a pre-processing step has been performed to clean, select and transform data to make them suitable for the analysis. First, we cleaned the collected data by removing all the points with unreliable positions (i.e., null coordinates and evident wrong values). Then, since infection data are spatially referenced at zip-code granularity, we transformed the trajectory data by assigning each pickUp and DropOff location to its specific Zip Code. In total, this matching step resulted in 23 Zip Codes (i.e., 60601, 60654, 60611, etc.) involved in the mobility data. The final dataset contains about 15,000 daily trajectories, each modeled as $<PickUp, DropOff>$ pairs and describing the set of trajectories traced by buses during a day. The total data size is about 28 MB. We report in the following the results of the analysis performed on such a dataset, by showing the zip codes of the city involved in the mobility data and the discovered mobility patterns.

**Mobility and Epidemic Patterns.** Mobility patterns (Cesario et al. 2017) have been discovered by applying the mlxtend.frequent-patterns Python library, a pattern mining implementation of the well known apriori algorithm. The discovered mining model is a set of mobility patterns describing sequential relations between the movement of the users under investigation. The number of mobility patterns extracted from the frequent regions highly depends on the minimum support. When the minimum support increases, the number of rules decreases. In our tests we set a support $s=0.6$ and we discovered 5 mobility patterns, involving 16 zip codes. In particular, the set of discovered mobility patterns is reported below:

$$EP_1$$:  60611,60654,60656,60661,60603  $$\rightarrow$$  60601

$$EP_2$$:  60654,60661,60601   $$\rightarrow$$   60603

$$EP_3$$:  60601,60603   $$\rightarrow$$   60611

$$EP_4$$:  60601,60603   $$\rightarrow$$   60661

$$EP_5$$:  60661,60603,60601   $$\rightarrow$$   60654

As the mobility patterns are detected, epidemic patterns are generated by selecting those ones having epidemic hotspots involved as both source and destination areas. As in our case each zip code region is also an epidemic hotspot, the five mobility patterns above listed are also epidemic patterns, whose antecedents and consequents will be further exploited to learn the epidemic forecasting models.

### Training and Testing the Forecasting Models

Having extracted the epidemic patterns, the next step is aimed at learning a specific epidemic forecasting model for each destination location, exploiting infection data of the source locations as regression variables. For such a reason, we perform now the training of five regression functions, one for each destination zip code: 60601, 60603, 60611, 60661, 60654.

As well known, to perform the regression task and its validation, we need to split the original dataset into two partitions: the training set and the test set. The first one is exploited to discover the relationships inside data while the second one is used for evaluating whether the discovered relationships hold. In our case, the overall infection data spans 21 months of data, and it has been split with respect to the number of months: the training set contains the infection data of the first 17 months (April 2020 - August 2021), while the test set holds the infection data of the last 4 months (September 2021 - December 2021). Thus, the training set contains the 81% of the data, while the test set the remaining 19%.

As described in the following sub-sections, we trained the knowledge models using data from April 2020 to August 2021 and we used the trained model to forecast the infection events from September 2021 to December 2021, to assess the quality of the predictions. Forecasting models have been discovered by applying the LSTM algorithm of the PyTorch library.

**Fig. 6:**
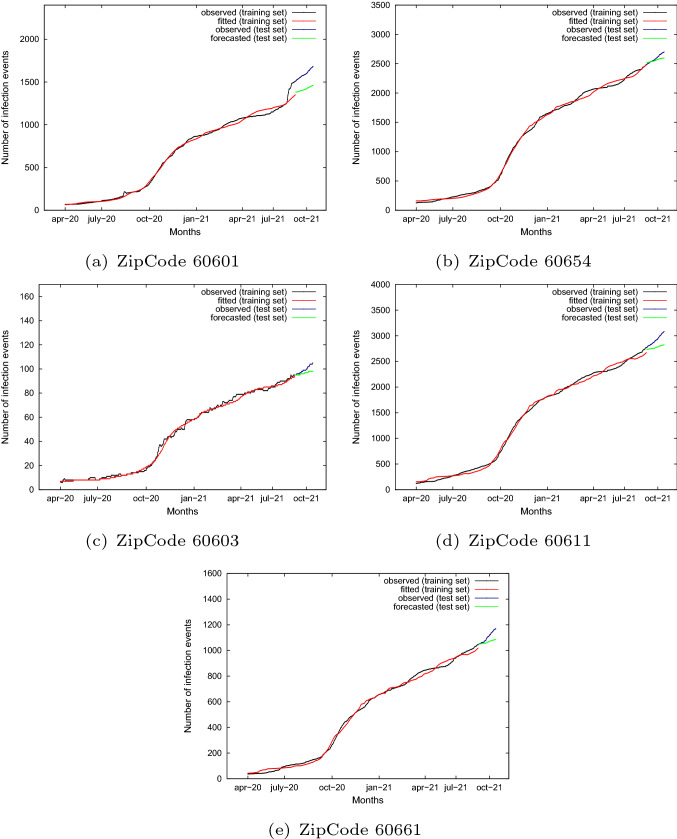
Cumulative number of infection events observed and fitted (black and red lines) on the training set, and cumulative number of infection events observed and forecasted (blue and green lines) on the test set, for different zip codes

To assess the effectiveness and accuracy of the regressive function modeled by LSTM regression model, we performed an evaluation analysis on the test set consisting of the last four months of data (i.e., from September 2021 to December 2021). In particular, the model has been used to predict future values of the number of positive cases that will occur in that area, day by day. The prediction of other types of associated events (i.e., number of hospitalized persons, number of deaths, etc.) is out of the scope of this work and it will be studied in a further research activity.

Let $y_i$ denote the $i{th}$ observation and $\widehat{y_i}$ denote the forecast of $y_i$ according to the LSTM model for $t=t_i$. Figure 6 shows the curves for the five zip codes under investigation. For the training set period, from April 2020 to August 2021, observed data and fitted data are plotted in black and red, respectively. For the test set period, i.e. from September 2021 to December 2021, observed and forecasted data are traced in blue and green, respectively. It is interesting to highlight that the regressive curve fits well the training data series. By looking at the test set, we can notice that forecasted data adhere very well to the observed data for that period. It is evident that the trend forecasted by the regressive model is very similar to that occurring in the observed data. In particular, we can notice that predicted values in general are a bit lower than observed data by showing an under-forecasting with respect to the real number of infection events.

### Experimental evaluation and comparative analysis

To make our evaluation more accurate and complete, we performed a comparative analysis of the proposed approach with an auto-regressive approach. In particular, auto-regressive (AR) models are a subset of time series models, which can be used to predict future values based on previous observations (AR models use regression techniques and rely on autocorrelation in order to make accurate predictions). AR models have been discovered by applying the AutoReg algorithm of the statsmodels.tsa.ar-model Python library.

Now, let us give a quantitative evaluation about the accuracy of the regressive model. To do that, we computed several indices (commonly used in the literature) to evaluate the forecasting accuracy. In particular, let $y_i$ denote the $i^{th}$ observation, $\widehat{y_i}$ denote the forecast of $y_i$ according the regressive model and ${\overline{y}}=mean(y)$, the indices are defined as follows:

- *Mean Absolute Error*: $MAE(y_i, \widehat{y_i})=\frac{1}{n} \sum _{i=0}^{n - 1}\mid y_i - \widehat{y_i}\mid$, i.e., a scale-dependent index measuring the average forecasting absolute error (the lower value, the better score);
- *Mean Absolute Percentage Error*: $MAPE(y_i, \widehat{y_i})=\frac{1}{n} \sum _{i=0}^{n - 1}\frac{\mid y_i - \widehat{y_i}\mid }{\mid y_i \mid }$, i.e., a scale-independent index computing the average forecasting percentage error (the lower value, the better score);
- *Mean Squared Error*: $MSE(y_i, \widehat{y_i})=\frac{1}{n} \sum _{i=0}^{n - 1} (y_i - \widehat{y_i})^2$, i.e., a general purpose error metric for numerical predictions that amplifies and severely punishes large errors (the lower value, the better score);
- *Median Absolute Error*: $MedAE(y_i, \widehat{y_i})=Median(\mid y_1 - \widehat{y_1}\mid , \dots , \mid y_n - \widehat{y_n}\mid )$, i.e., a scale-dependent index measuring the median absolute forecasting error, which is particularly interesting because it is robust to outliers (the lower value, the better score);
- $R^2$ *score*: $R^2(y_i, \widehat{y_i})=1 - \frac{\sum _{i=1}^{n} (y_i - \widehat{y_i})^2}{\sum _{i=1}^{n}(y_i - {\overline{y}})^2}$, i.e., a general purpose error metric for numerical predictions that represents the proportion of variance (of y) that has been explained by the independent variables in the model. It provides an indication of goodness of fit and therefore a measure of how well unseen samples are likely to be predicted by the model, through the proportion of explained variance. The best score is 1.0 and it can be negative because the model can be arbitrarily worse (the higher value, the better score);
- *Explained Variance Score*: $EVS(y_i, \widehat{y_i})=1 - \frac{Var(y - {\widehat{y}})}{Var(y)}$, which measures the proportion to which a mathematical model accounts for the variance (dispersion) of a given data set, whose the best possible score is 1.0 (the higher value, the better score).

**Fig. 7:**
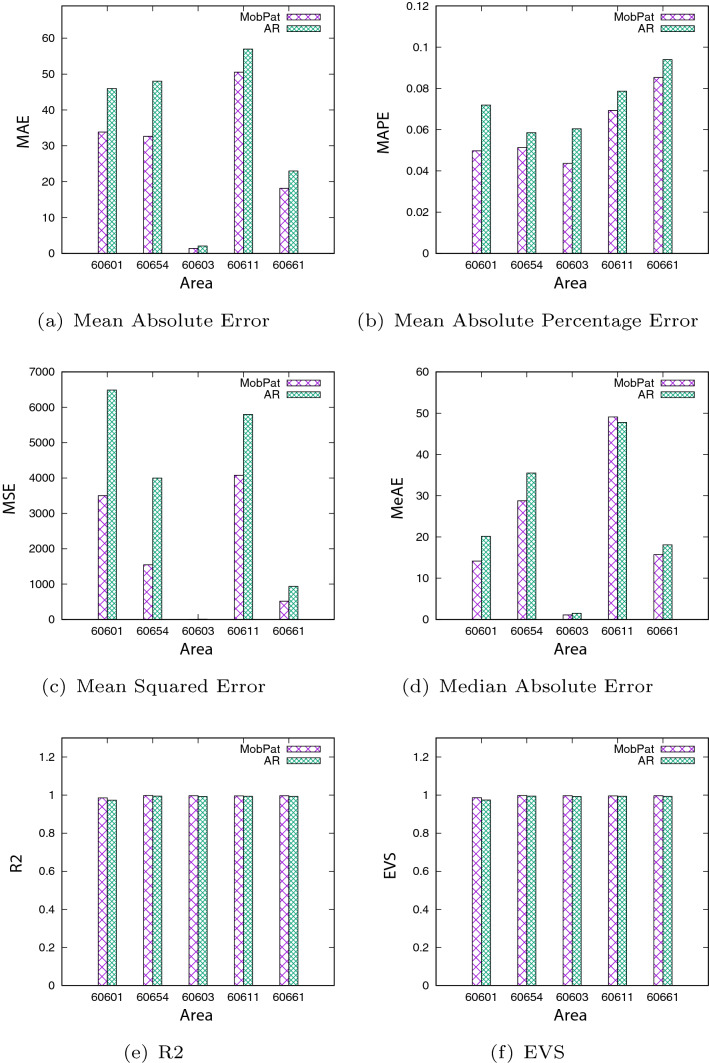
Comparative analysis among the two approaches, evaluating six indices, for the zip-codes under investigation

To perform the comparative analysis, we evaluated the forecasting performance of the two approaches on the test set of the five areas (identified by the zip-codes). The results for the algorithms were obtained by performing an accurate tuning of the input parameters: for each dataset, different runs were executed for different values of the parameters, then the best results were selected. The results shown below only refer to the run with the best combination of parameters. Figure 7 summarizes the results of the comparison between our proposed approach based on mobility patterns (MobPat) and the auto-regression approach based only on a auto-regressive formula (AR). The figure shows the achieved indices, for the five areas (zip-codes) under investigation. In particular, we can see that our approach largely achieves better performances than other algorithms, in terms of MAE, MAPE, MSE and MedAE, for all five zip-codes (but MedAE on the zip-codes 60611, as shown in Fig. 7d). Taking into account the two indices $R^2$ and *EVS*, even if it is less evident, our approach results in better performance compared to the other. These results confirm the appropriateness of the proposed approach based on mobility patterns and its good performance in the epidemic prediction domain.

## Conclusion

COVID-19 has been resulting as one of the worst pandemics in history, which has been responsible for more than four hundred million reported cases. This has motivated a research effort towards the study of data-driven predictive models for epidemic events, whose effectiveness is crucial to support decision-makers in the efficient management and utilization of healthcare resources.

This paper presented the design and implementation of an approach based on spatial analysis and regressive models to discover spatio-temporal predictive epidemic models from mobility and infection data. First, the algorithm extracts infection hotspots, mobility hotspots, and mobility patterns. Then, on the basis of such knowledge, as infectious diseases are mainly spread through human-human transmissions, epidemic patterns are extracted from the subset of mobility patterns involving epidemic hotspots. Finally, the approach extracts a specific epidemic forecasting model for each epidemic hotspot, by analyzing the infection data. The experimental evaluation, performed on a real-world data set collecting the infection cases of some areas of Chicago, showed that the proposed methodology can forecast the number of positive cases with good accuracy. Furthermore, we also presented a comparative analysis with an auto-regressive algorithm exploited as base-line.

In future work, other research issues may be investigated. First, we may perform an extended experimental evaluation on other urban territories, to assess the results obtained in the case study reported here. Second, we may further explore the application of other spatial analysis approaches and regressive algorithms for the extraction of forecasting models. Third, in addition to the number of positive cases, it may be interesting to investigate some methodologies that predict other relevant indicators related to the epidemics under investigation.

## Footnotes

1 https://observablehq.com/@chicagoreporter/historical-illinois-covid-19-zip-code-data?collection=@chicagoreporter/illinois-coronavirus-data

2 https://observablehq.com/
